# A Passage of Time Signal in the Human Brain

**DOI:** 10.1523/ENEURO.0406-25.2025

**Published:** 2026-01-15

**Authors:** Virginie van Wassenhove, Benjamin R. Kanter, Simone Viganò, Raphaël Bordas

**Affiliations:** ^1^CEA, DRF/Joliot, NeuroSpin, INSERM, Cognitive Neuroimaging Unit, Université Paris-Saclay, Gif/Yvette F-91191, France; ^2^Kavli Institute for Systems Neuroscience and Centre for Algorithms in the Cortex, Norwegian University of Science and Technology, Trondheim 7030, Norway; ^3^Department of Psychology, Max Planck Institute for Human Cognitive and Brain Sciences, Leipzig 04103, Germany; ^4^Center for Mind/Brain Sciences, University of Trento, Rovereto 38068, Italy

In a dense-sampling resting–state functional magnetic resonance imaging study, Wang et al. (2025) recorded two individuals’ functional connectivity patterns over 30 consecutive days to find a marker of the passage of time in the human brain. The authors measured the similarity of brain connectivity patterns over days, focusing on key regions involved in spatial navigation and declarative memory that have been previously shown to exhibit slow changes in activity patterns over time: the entorhinal cortex (EC) and the hippocampus (HPC). The authors show that connectivity pattern similarity decreased over time—more temporally distant resting-states had more distinct functional connectivity profiles. This result is consistent with the idea that brain activity intrinsically drifts over time (Driscoll et al., 2022). Additionally, the authors observed an anatomical gradient such that the anterior HPC showed stronger temporal drift than the posterior HPC, and the anterolateral EC showed stronger temporal drift than the posteromedial EC. The temporal drift of the EC whole-brain functional connectivity pattern was primarily driven by the default mode network, typically reported when participants are not engaged in any experimental task. The authors conclude that the human brain maintains an intrinsic temporal context signal that may provide “time stamps” for episodic memories, helping to organize events in time.

One open question concerns the authors’ choice to quantify the drift using “functional connectivity patterns” rather than “within-region multivoxel pattern similarity” over time (Bellmund et al., 2019). If temporal drift in neural representations truly reflects a “time stamp” signal, one might expect it to manifest most directly in the evolving activity patterns within the HPC and EC themselves, a form of intrinsic dynamics that could be seen as a continuously “rolling” neural trace of time. In contrast, connectivity drift may capture how these intrinsic changes propagate to or are integrated across cortical systems, potentially reflecting a distinct but complementary aspect of temporal coding, such as the integration of incoming external information to be assigned to the time stamps or the consolidation of temporally distinct memories into the neocortex.

From a cross-species point of view, the increasing drift over time observed in HPC and EC aligns well with rodent studies in which hippocampal and entorhinal neural populations have been shown to encode time. For example, individual neurons are active at specific times when animals perform a task with repeating temporal structure (so-called time cells; Eichenbaum, 2014; Tsao et al., 2022). Moreover, at the single-cell and population level, neural activity in these areas slowly drifts over time from seconds to minutes to days, a signal seen most prominently in the lateral EC (Tsao et al., 2018; Kanter et al., 2025). Such drift over time is independent of the specific task or experience, continuing even during sleep (Kanter et al., 2025). The recent findings from Wang et al. (2025) fit nicely with this idea that the (antero)lateral EC automatically encodes the passage of time across a wide range of timescales, consistent with the demands of episodic memory. By analogy to the varied scales of spatial representation (Brunec, 2016), the authors report an anteroposterior HPC gradient with stronger drift in the anterior portion, suggesting a potential change from finer to coarser temporal granularity.

In addition to drifting over time, recent work in rats (Kanter et al., 2025) and humans (Ezzyat and Davachi, 2014; Ben-Yakov and Henson, 2018; Clewett et al., 2019; Zheng et al., 2022) found that neural activity in the hippocampal formation signals event boundaries, segmenting a continuous experience into discrete temporal units. It would have been interesting in the current study to ask whether the drift signal could be parsed into distinct temporal scales: are days and scan durations tagged in spontaneous activity? In humans, days provide natural event boundaries that create distinct episodes in memory. Days serve as reliable access points and time stamps forming a hierarchical temporal structure in human memory captured by the behavioral day-of-the-week effect (Wagelmans and van Wassenhove, 2024), in which participants are faster in responding which day it is on weekends as compared with weekdays. Wang et al. (2025) used continuous similarity measures to quantify the drift across daily sessions but whether days mark the passage of time in humans could also be explored using the within-region multivoxel pattern similarity suggested above. Additionally, the drift was observed using temporally structured recordings: while one participant had a fixed recording schedule (daily at 11 A.M.), the other underwent a change of recording schedule (in periods of 10 d, at 7 A.M., then 7 A.M. and 8 P.M., and finally 8 P.M.). The within-day changes in the recording schedule of the second participant showed no modulation of the drift. In light of the anatomical gradient reported by the authors, anterior HPC and EC could have been expected to track within-day drift hierarchically nested into wider temporal drift in the posterior portions. This result would strengthen the functional relevance of the temporal gradient reported by the authors.

The authors strongly conclude that they have found “a spontaneous neural signature that reflects the passage of time in humans in the absence of task demands, which may serve to provide temporal stamps for episodic memory processes.” One positive aspect of their approach is that participants do not attend to time, that is, they do not a priori deploy cognitive strategies to keep track of time (e.g., counting, simulating, internal speech) or explicitly encode temporal experiences in memory. This makes the study amenable to episodic timing in which changes in the “what,” “where,” and especially “when” of experiences are automatically tracked in the absence of task requirements (Azizi et al., 2023; Bordas and van Wassenhove, 2025). However, one major limitation of the study is the absence of insights on participants’ subjective experiences. Although the authors convincingly demonstrate spontaneous neural drift as a function of time, they do not directly link it to the subjective experience of time flow or to memory recall. For instance, is the reported signature of the passage time related to time awareness? Would the drift be more similar across participants who experience the passage of time similarly? A key challenge in designing tasks or questionnaires to measure participants’ subjective experience of time passing is that their cognitive approach or temporal focus during the task could influence the memory consolidation processes reflected in functional connectivity patterns. This concern becomes particularly salient when the same participant completes multiple scans over time, as in the current design. One alternative approach would involve keeping participants unaware of any time-related task during the recording session and then asking them afterward to estimate how much time has passed. This approach was recently tested (Azizi et al., 2023; Bordas and van Wassenhove, 2025): participants recorded with MEG or EEG during quiet wakefulness for a few minutes were unexpectedly prompted to recall how long the experiment lasted, in retrospect. In these studies, the automatic (unattended) duration of a resting-state episode was found to be linearly related to the relative time of oscillatory alpha activity (or burstiness) during the recorded period. This relationship was broken if participants developed temporal expectations in the course of the experiment (Bordas and van Wassenhove, 2025). Hence, in Wang et al. (2025), one possibility would have been to ask for a single retrospective passage of time judgment. Still, after which session this report should be placed remains tricky: asking a retrospective time report at the end of the first session would ensure the cleanest time estimate but risks altering subsequent memory consolidation processes; placing the report at the end of the last session would prevent self-awareness of time passing from altering the neural drift but will also alter the nature of the introspective report, which will become context-dependent (e.g., estimating the duration or felt speed of time in the last session with respect to the mean of all previous sessions; Bordas and van Wassenhove, 2025).

In sum, the work by Wang et al. (2025) nicely echoes the conclusions recently put forth in a series of studies (Azizi et al., 2023; Bordas and van Wassenhove, 2025), which directly links participants’ retrospective reports of elapsed time with resting-state brain dynamics over a timescale of minutes. These findings are very exciting by suggesting possible direct links between the flow of thoughts, time, and memory. Future work should test whether this neural signal supports the encoding of temporal information or the construction of temporal awareness. It is about time for these fields of research to bridge our understanding of how the brain maps time (Kwok et al., 2025).

## Synthesis

Reviewing Editor: Niko Busch, Westfalische Wilhelms-Universitat Munster Decisions are customarily a result of the Reviewing Editor and the peer reviewers coming together and discussing their recommendations until a consensus is reached. When revisions are invited, a fact-based synthesis statement explaining their decision and outlining what is needed to prepare a revision will be listed below. The following reviewer(s) agreed to reveal their identity: Joost de Jong.

# Review synthesis:

The reviewer provided a very positive evaluation of the commentary, praising its clarity and its success in highlighting both the significance and future directions of the original study. They raised one optional point for consideration, namely, that the authors could contextualize their remark about the absence of participant reports by acknowledging that adding such reports may itself alter functional connectivity patterns.

I encourage the authors to consider this suggestion and, if they agree, briefly discuss why the inclusion of behavioral reports may not be straightforward in this context.

Additionally, when praising the original study in the sentence "One positive aspect of their approach is that participants do not a priori attend to time, making the recording an episodic timing event", the authors could elaborate on what makes this feature so particularly valuable and why it matters.

# Reviewer 1

I have read the commentary about a 'passage-of-time signal in the human brain' with great interest. It made me eager to read the original article. The authors were able not only to distill the work's essential contribution to the literature but also to point to fascinating ways that this line of research can be extended.

I only have one suggestion for something that may be added, but the editor can decide whether that is necessary at all. The authors point to the absence of reports in the original study as a drawback. But, given that they are potentially looking at memory consolidation in neocortex, reporting recalled memories may significantly change functional connectivity patterns going forward. I do agree that reports are necessary to link these neural drifts to temporal memory, but I also think that adding reports is not as straightforward as it may seem at first glance. Maybe you'd need a design where some events are prompted for recall but not others? Hence, this potential drawback may be contextualized as such. But again, I will leave that to the editor, since I believe that the commentary is great as is.
